# Poly(3-hydroxybutyrate) Degradation by *Bacillus infantis* sp. Isolated from Soil and Identification of *phaZ* and *bdhA* Expressing PHB Depolymerase

**DOI:** 10.4014/jmb.2303.03013

**Published:** 2023-05-29

**Authors:** Yubin Jeon, HyeJi Jin, Youjung Kong, Haeng-Geun Cha, Byung Wook Lee, Kyungjae Yu, Byongson Yi, Hee Taek Kim, Jeong Chan Joo, Yung-Hun Yang, Jongbok Lee, Sang-Kyu Jung, See-Hyoung Park, Kyungmoon Park

**Affiliations:** 1 Department of Biological and Chemical Engineering, Hongik University, Sejong 30016, Republic of Korea; 2 Department of Food Science and Technology, Chungnam National University, Daejeon 34134, Republic of Korea; 3 Department of Biotechnology, The Catholic University of Korea, Bucheon 14662, Republic of Korea; 4 Department of Biological Engineering, Konkuk University, Seoul 05029, Republic of Korea

**Keywords:** Poly(3-hydroxybutyrate), biodegradation, *Bacillus infantis*, PHB depolymerase, *phaZ*, *bdhA*

## Abstract

Poly(3-hydroxybutyrate) (PHB) is a biodegradable and biocompatible bioplastic. Effective PHB degradation in nutrient-poor environments is required for industrial and practical applications of PHB. To screen for PHB-degrading strains, PHB double-layer plates were prepared and three new *Bacillus infantis* species with PHB-degrading ability were isolated from the soil. In addition, *phaZ* and *bdhA* of all isolated *B. infantis* were confirmed using a *Bacillus* sp. universal primer set and established polymerase chain reaction conditions. To evaluate the effective PHB degradation ability under nutrient-deficient conditions, PHB film degradation was performed in mineral medium, resulting in a PHB degradation rate of 98.71% for *B. infantis* PD3, which was confirmed in 5 d. Physical changes in the degraded PHB films were analyzed. The decrease in molecular weight due to biodegradation was confirmed using gel permeation chromatography and surface erosion of the PHB film was observed using scanning electron microscopy. To the best of our knowledge, this is the first study on *B. infantis* showing its excellent PHB degradation ability and is expected to contribute to PHB commercialization and industrial composting.

## Introduction

Plastics are widely used in our daily lives and industries because of their excellent mechanical strength and thermal stability [1]. As the amount of plastic used increases, environmental pollution due to landfilling and incineration of plastic waste has become a problem. As an alternative for waste treatment and pollution, research on biodegradable plastics that can be degraded by microorganisms is being actively conducted [2, 3]. Among these bioplastics, poly(3-hydroxybutyrate) (PHB), which is the most representative structure of polyalkanoates (PHAs), is biosynthetic and biodegradable only through microbial processes, and has high human body compatibility and potential applications in the medical field [4]. However, owing to the low biodegradability of biodegradable plastics, it is impossible to recycle them, and they are discharged as general garbage rather than as separate discharges [5]. Therefore, to commercialize biodegradable plastics, a method that can effectively degrade them within a short time is required [6, 7].

Microorganisms known to degrade PHB include *Bacillus* spp., *Microbulbifer* spp., *Ralstonia eutropha*, and the genus Streptomyces [8-10]. *B. infantis* among *Bacillus* spp. are found in various environments such as soil and sea, and have the ability to degrade azo dyes that are difficult to biodegrade [11]. There are reports of strains known to have PHB-degrading abilities, but there are no reports on the specific activity and characteristics of these PHB-degrading strains [8]. Biodegradation occurs easily when PHB is the only carbon source in an environment with limited nitrogen and carbon sources [12]. Therefore, for effective PHB biodegradation, it is important to identify strains with excellent PHB degradation abilities under nitrogen- and carbon-limited conditions.

The PHB degradation pathway consists of the hydrolysis of PHB to the monomer 3-hydroxybutyrate by PHB depolymerase (PhaZ) and the conversion of 3-hydroxybutyrate to acetoacetate by 3-hydroxybutyrate dehydrogenase (BdhA), which is used for microbial metabolism [13, 14]. These two key enzymes are regulated by the *phaZ* and *bdhA* genes, respectively [15]. Therefore, the PHB degradation ability can be inferred from the identification of microorganisms with *phaZ* and *bdhA* and further improvements in PHB degradation ability can be expected through gene cloning and overexpression [16].

In this study, we isolated microorganisms that effectively degraded PHB from soil. A halo assay was performed to examine the PHB degradation ability of the isolated microorganisms in a PHB double-layer plate [17]. The *phaZ* and *bdhA* genes of the PHB-degrading enzymes in *B. infantis* were identified using polymerase chain reaction (PCR) in *B. infantis*. We aimed to measure the PHB film degradation rate in a mineral-rich medium with a limited nitrogen source under soil-like conditions and confirmed the excellent PHB-degrading ability of the isolated strain in mineral medium (MM) [17]. The molecular weight changes and surface degradation of the PHB films were analyzed. Additionally, its degradability against other bioplastics was tested. Novel strains with excellent PHB degradation abilities and the identification of *phaZ* and *bdhA* using PCR suggest the possibility of commercializing PHB and its industrial benefits for effective PHB degradation.

## Materials and Methods

### Chemicals

All chemicals used in this study were of analytical grade. The chloroform, PHB powder, and PHB films used for the plates and degradation experiments were purchased from Sigma-Aldrich (USA). The PHB pellets used for gel permeation chromatography (GPC) analysis were purchased from Goodfellow (UK).

### Isolation of PHB-Degrading Strains

Soil samples were collected from a depth of 10 cm below the surface of a garbage landfill in Sejong, Korea and rice fields in Suwon, Korea. Each soil sample (1 g) was diluted in 10^-1^, 10^-2^, or 10^-3^ autoclaved distilled water (DW) and spread onto Luria-Bertani (LB) agar plates. The plates were incubated at 37°C for 1 d, and colonies with different morphological characteristics were isolated from each plate. The colonies were incubated in liquid medium for 1 d to prepare stocks containing 20% (w/v) glycerol and were stored at -70°C until further use.

### Halo Assay: Bioplastic Double-Layer Plate

To screen for the PHB degradation strain, 10 g/l PHB powder was suspended in DW and autoclaved at 121°C for 15 min. After autoclaving, the PHB powder suspension was stirred overnight at 150–180 rpm. Autoclaved agar medium (20 ml) was then added to the plate. The PHB suspension was mixed with autoclaved medium containing 2% agar at a ratio of 1:1 and poured onto the top layer [17]. Autoclaved paper discs were placed in the specific section of the PHB double layer plate and inoculated with 8 μl of culture medium. Cultivation was performed at 37°C for 1 d. To prepare suspensions of other bioplastics such as polybutylene adipate terephthalate (PBAT) and polybutylene succinate (PBS), 0.2 g of bioplastics were emulsified in 30 ml of dichloromethane (DCM). Next, 100 ml of DW was added and sonicated for 10 min using a VC 505 (Sonics & Materials, Inc., USA). After sonication, the solution was heated in a 60°C water bath to evaporate DCM and autoclaved at 121°C for 15 min [18].

### 16S rRNA Sequencing

Colonies forming halo zones on the PHB plates were identified at the species level using 16S rRNA sequencing, PCR amplification, and the primers 27F and 1492R. Partial sequences were obtained using Solgent (Korea) and compared to sequences in the National Center for Biotechnology Information (NCBI) GenBank database (https://blast.ncbi.nlm.nih.gov/Blast.cgi) using BLASTN tools [19].

### Identification of PHB Degradation Genes Using PCR

To identify *phaZ* and *bdhA* in strains with PHB degradation activity, universal primers for *Bacillus* species were designed for each gene. MEGA-X was used to align *phaZ* sequences from 29 *Bacillus* strains and *bdhA* sequences from 35 *Bacillus* strains. Among the aligned sequences, primers were designed by identifying the conserved regions common to most strains. Primer sequences used to identify *phaZ* using PCR were forward (5’-AATAAGTGTTGGAACTGGTTTGA-3’) and reverse (5’-GTGTCGGTATTGTACATTCCTTTATC-3’). The primer sequences used to identify *bdhA* by PCR were forward (5’-ATTGAAGAATTTCCTACAGAA-3’) and reverse (5’-TGGAGTATCCACATAACCAGGGCA-3’). The PCR conditions for *phaZ* and *bdhA* used in this study are listed in Table 1. The polymerase used for PCR was 2X Pfu PCR Smart Mix 1 (Solgent, Korea). PCR products were separated using electrophoresis at 100 mV for 30 min on 1.5% agarose gel containing 4 μl EcoDye DNA Staining Solution (Solgent) in TAE buffer (Bioneer, Korea).

### PHB Film Degradation Analysis

*Bacillus algicola* SOL02 strain provided by Konkuk University (Professor Yung-Hun Yang) was used as a control [19]. Before cultivating strains with PHB, pre-culture was performed in tryptic soy broth (TSB; 1.7% tryptone, 0.3% soytone, 0.25% glucose, 0.5% NaCl, and 0.25% dipotassium phosphate) at 37°C for 1 d. PHB films (0.2 g/l) were autoclaved at 121°C for 15 min and incubated in 100 ml of liquid medium with 5 ml pre-cultured broth at 37°C for 5 d. Cultivation was performed in a shaking incubator at 200 rpm. The composition of mineral medium (MM) was referenced from Leibniz Institute web-site (DSMZ-German Collection of Microorganisms and Cell Cultures) and is as follows: 2.44 g/l Na_2_HPO_4_, 1.52 g/l KH_2_PO_4_, 0.5 g/l (NH_4_)_2_SO_4_, 0.2 g/l MgSO_4_·7H_2_O, 0.05 g/l CaCl_2_·2H_2_O, and 10 ml/l trace element solution SL-4 (trace element solution SL-4:0.5 g/l EDTA, 0.2 g/l FeSO_4_·7H_2_O, trace element solution SL-6 100 ml/l (trace element solution SL-6: 0.3 g/l H_3_BO_3_, 0.2 g/l CoCl_2_·6H_2_O, 0.02 g/l NiCl_2_·6H_2_O, 0.03 g/l Na_2_MoO_4_·2H_2_O)). After cultivation, the PHB films were collected, washed with DW, and dried in an oven for 2 d.

### Analysis of Physical Changes on the Surface of PHB Film

To observe surface changes on the PHB film after degradation, scanning electron microscopy (SEM) was used. The samples were collected after incubation, washed with DW, and dried in an oven. Then, the PHB sample was coated with gold at 5 mA for 120 s, and backscatter electron images were acquired using SEM (JSM-IT700HR, USA, Jeol) at an accelerating voltage of 3 kV.

### Analysis of Molecular Weight Reduction of PHB Film

GPC (YL Chromass, Korea) was performed to determine the molecular weight and mass distribution of PHB. For GPC analysis, 1 g/l PHB pellets were emulsified in chloroform at 60°C for 2 h. Chloroform was evaporated in a fume hood until a plastic film formed. The PHB films were collected after incubation, washed with DW, and dried in an oven. For sample preparation, the PHB film was dissolved in chloroform at 60°C for 1 h. This solution was filtered through a 0.2-μm pore size syringe filter (Chromdisc, Korea) to separate the dissolved PHB from the remaining insoluble components. A high-performance liquid chromatography (HPLC) system consisting of a loop injector (Rheodyne 7725i), an isocratic pump with dual heads (YL9112), a column oven (YL9131), columns (Shodex, K-805, 8.0 I.D. × 300 mm, Shodex, K-804, 8.0 I.D. × 300 mm), and an refractive index (RI) detector (YL9170) was used for analysis. Sixty microliters of the solution without air bubbles were injected. Chloroform was used as the mobile phase at a flow rate of 1 ml/min and a temperature of 40°C. The data were analyzed using the YL-Clarity software for a single YL HPLC instrument (YL Chromass). Molecular masses were analyzed in relation to polystyrene standards ranging 5000–2,000,000 g/mol [21].

## Results and Discussion 

### Isolation of *B. infantis* Strains

**Fig. 1:**
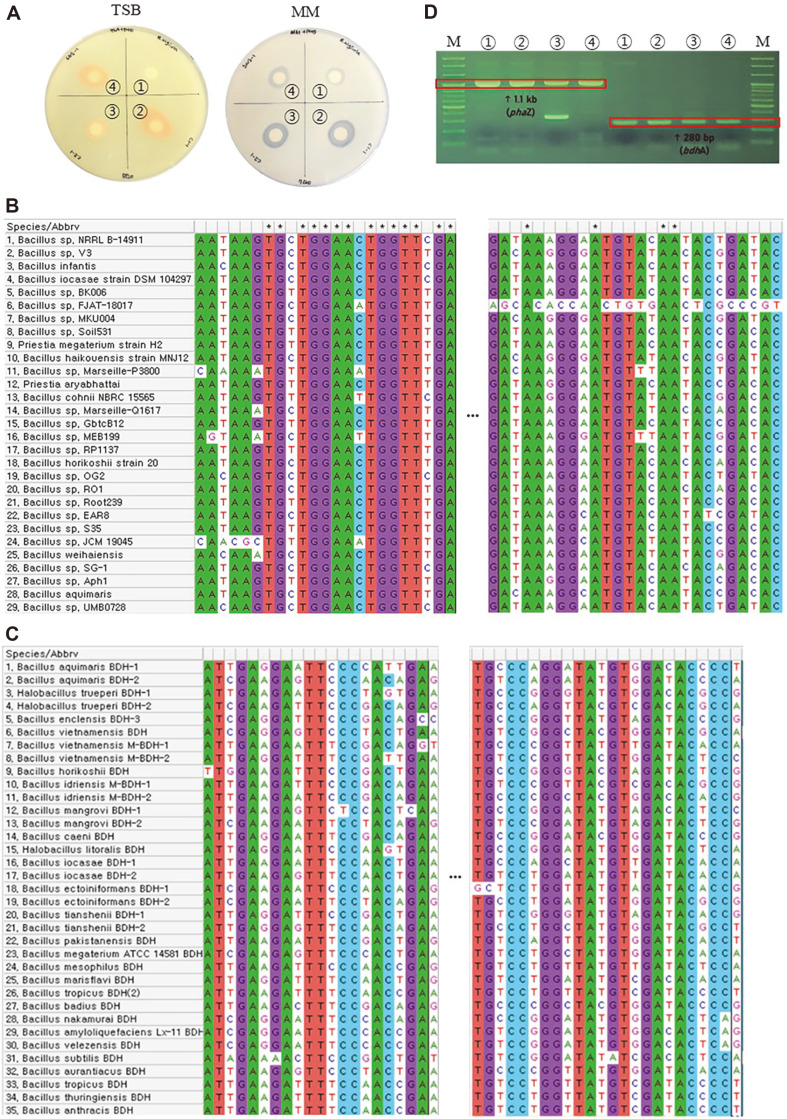
Screening of PHB-degrading bacteria and genes. M:100 bp DNA ladder. (1) *B. algicola* SOL02, positive control. (2) *B. infantis* PD1. (3) *B. infantis* PD2. (4) *B. infantis* PD3. (**A**) Halo assay result on TSB medium and MM for 1 d. (**B**) Forward and reverse primer section for *Bacillus* spp. *phaZ* identification. (**C**) Forward and reverse primer section for *Bacillus* spp. *bdhA* identification. (**D**) Identification of PHB-degrading enzymes using PCR.

PHB-degrading bacteria were isolated from soil samples from landfills in Sejong, Korea, and rice fields in Suwon, Korea, where plastic waste was buried and generated. Soil samples were collected from a depth of 10 cm below the soil surface. After diluting the collected samples, various strains were isolated based on their morphological characteristics, such as colony size and color. A halo assay was performed to test the ability of the isolated strains; three strains showed the fastest degradation rate. By analyzing the 16S rRNA of the three strains that formed the halo zone, it was confirmed that all three strains were *B. infantis* with high similarity (homology 100, 99.9, and 100% for *B. infantis* PD1, PD2, and PD3, respectively), and were named *B. infantis* PD1, *B. infantis* PD2, and *B. infantis* PD3. They were deposited at the Biological Resources Center of the Korea Research Institute of Bioscience and Biotechnology under the accession numbers KCTC 19079P, KCTC 19080P, and KCTC 19081P, respectively. Interestingly, no halo zone was formed in TSB with a nitrogen source (1.7% tryptone and 0.3%soytone), but a distinct halo zone was formed in MM without a nitrogen source (Fig. 1A). To screen for PHB-degrading bacteria, strains are typically cultured on PHB-containing agar plates for 3–7 d, or more than 3 weeks [8, 17, 22]. In this study, all three *B. infantis* strains isolated from soil formed a clear halo zone within 1 d, and the PHB double-layer plate was effective for the PHB halo assay.

### Identification of *phaZ* and *bdhA* in *B. infantis* Using Universal Primers

PhaZ and BdhA are involved in PHB degradation pathway [15]. PhaZ hydrolyzes PHB to 3-hydroxybutyrate, and BdhA converts 3-hydroxybutyrate to acetoacetate. These two important PHB-degrading enzymes in *B. infantis* PD1, PD2, and PD3, were identified using PCR with a *Bacillus* species-specific universal primer set and optimized PCR conditions. Primer sets used to identify the *phaZ* and *bdhA* genes of each enzyme were established by searching the nucleotide sequences of *Bacillus* species in the NCBI database. Primer sets were designed to amplify the conserved regions of 1.1 of 1.77 kb for *phaZ* and 280 of 780 bp for *bdhA*. The *phaZ* conserved region for amplification using PCR is relatively long; therefore, stable and reliable *phaZ* detection can be expected [23]. The primers were designed by aligning the sequences of *Bacillus* species in the NCBI database using MEGA-X software (Figs. 1B and 1C). Additionally, we developed a method to identify *phaZ* and *bdhA* at the gene level. The presence of *phaZ* and *bdhA* in all three strains was confirmed using electrophoresis (Fig. 1D). Sequencing revealed that the nucleotide sequences of each band obtained after electrophoresis in the three strains were consistent with those of *phaZ* and *bdhA* in *B. infantis* registered in the NCBI database. Therefore, *B. infantis* PD1, PD2, and PD3 are considered to have both types of genes encoding the key enzymes that degrade PHB.

### Analysis of PHB Film Degradation in Liquid Medium

**Fig. 2:**
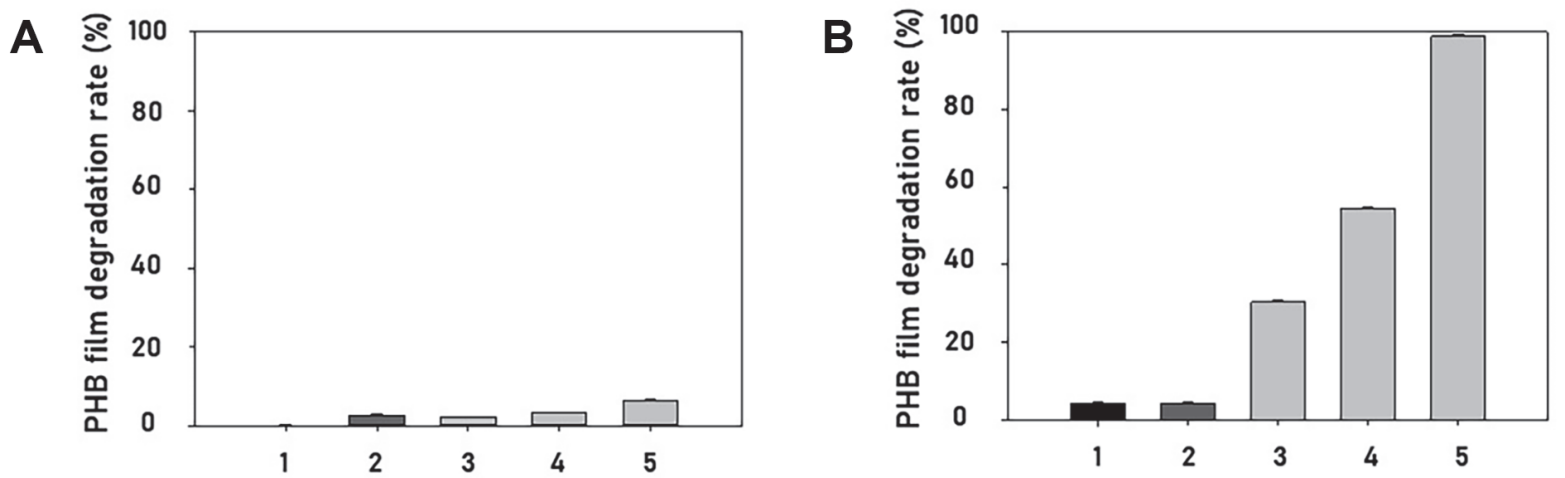
Degradation rate (%) of PHB film by *B. infantis*. PHB films were incubated in liquid mediums with no-cell (1), *B. algicola* SOL02 (2), *B. infantis* PD1 (3), *B. infantis* PD2 (4), and *B. infantis* PD3 (5) at 37°C for 5 d. (**A**) TSB, an optimal culture medium of *B. infantis*. (**B**) MM without nitrogen source. Each bar represents the mean ± SD (standard deviation) of three independent experiments.

**Fig. 3:**
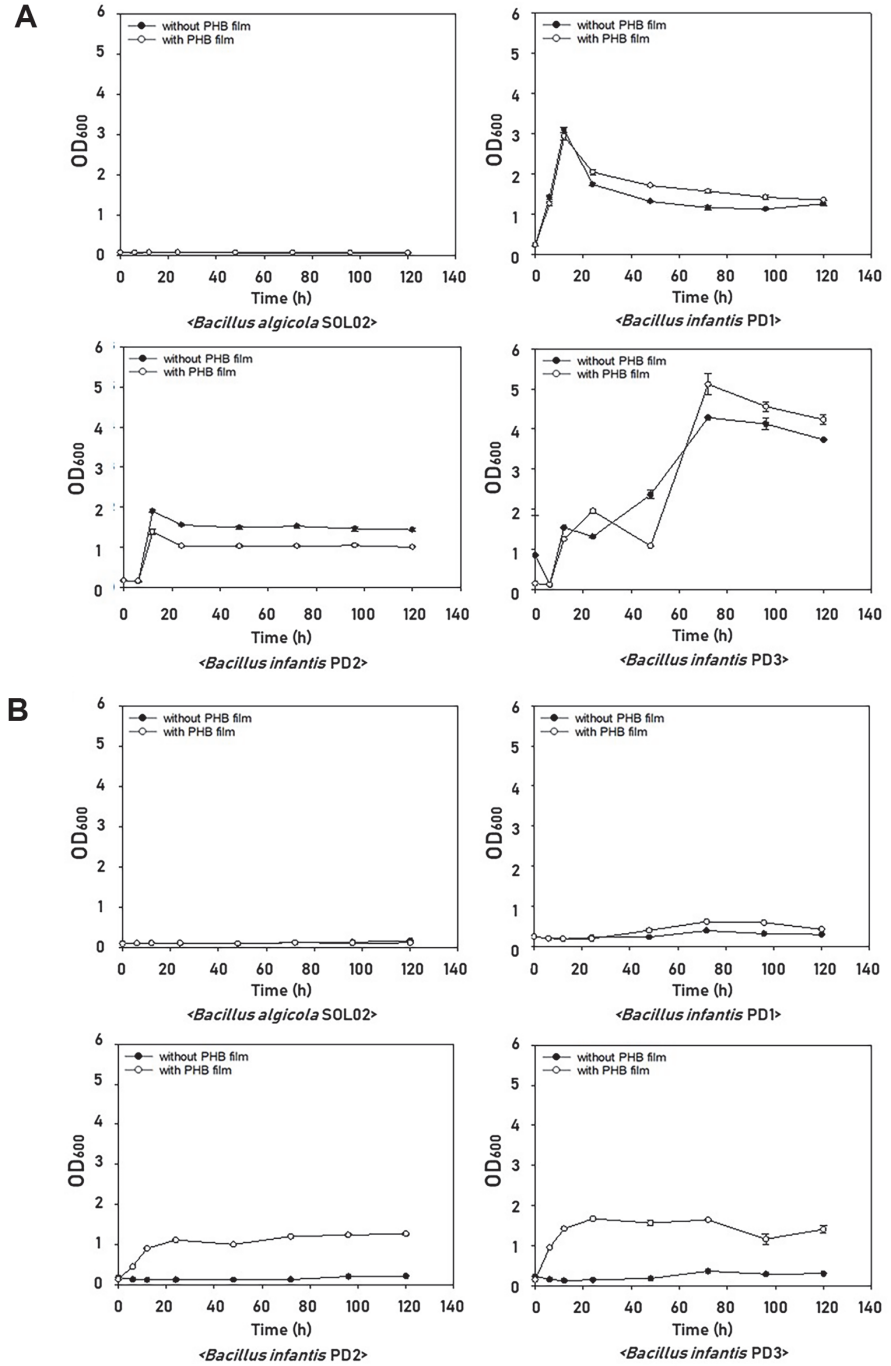
Cell growth curves of *B. infantis* by medium compositions with or without PHB film. Strains were cultured for 5 d in conditions with or without PHB. (**A**) TSB. (**B**) MM. Each bar represents the mean ± SD of three independent experiments.

PHB is used as a carbon source for microorganism metabolism and is known to be degraded under conditions where nitrogen sources are limited. Therefore, TSB rich in nitrogen and MM without nitrogen were used to determine which medium was suitable for PHB degradation. TSB is the optimal medium for the growth of *B. infantis*. *B. infantis* PD1 and PD2 exhibited stable growth curves in TSB (Fig. 3A); however, their PHB film degradation rates were only 2.31% and 3.19%, respectively (Fig. 2A). *B. infantis* PD3 showed the highest optical density (OD)_600_ and PHB film degradation rate (6.39 %) in TSB. The same experiment was performed by limiting the nitrogen source in the medium compositions. Compared to TSB, the growth of *B. infantis* PD1, PD2, and PD3 decreased in MM without PHB film (Fig. 3B). The PHB film degradation rate was 4.25% for *B. algicola* SOL02, 30.29% for *B. infantis* PD1, 54.41% for *B. infantis* PD2, and 98.71% for *B. infantis* PD3 after 5 d (Fig. 2B). In *B. infantis* PD3, most of the PHB film was degraded within 5 d under a limited nitrogen source. In the case of *B. algicola* SOL02, which is known to degrade PHB, growth was not observed, which was expected because TSB is not an appropriate medium for the growth of *B. algicola* SOL02 [8]. The growth of the three *B. infantis* PD1, PD2, and PD3 strains was reduced in MM because the nitrogen source required for bacterial growth was insufficient. However, the growth of all three *B. infantis* improved when the PHB film was present, indicating that the isolated and selected *B. infantis* can effectively degrade PHB under conditions containing various minerals using PHB as a carbon source for bacterial growth. In particular, *B. infantis* PD3 can be cultured in a limited nitrogen environment and has an excellent PHB degradation ability. Previous studies have shown that PHB degradation is induced and promoted under stressful conditions such as nitrogen limitation and poor nutrition [24, 25]. Consistent with previous reports, in the present study, PHB degradation was effectively induced by providing PHB as the sole carbon source and limiting nitrogen, which can stress the strains. Because these advantages are economically and environmentally suitable for the treatment of biodegradable plastic waste, an economical and eco-friendly approach can be expected using *B. infantis* PD3 in an industrial composting facility to reduce the cost and time required for processing biodegradation [26].

### Analysis of Physical Properties of Degraded PHB Film

**Fig. 4:**
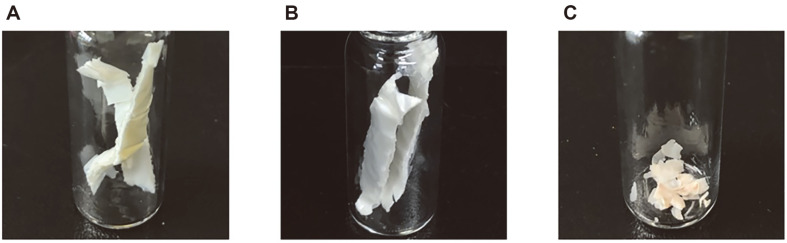
PHB film following degradation for physical analysis. PHB film was recovered after 5 d of incubation, washed with DW, and dried. (**A**) Before degradation. (**B**) After degradation in TSB. (**C**) After degradation in MM.

**Fig. 5:**
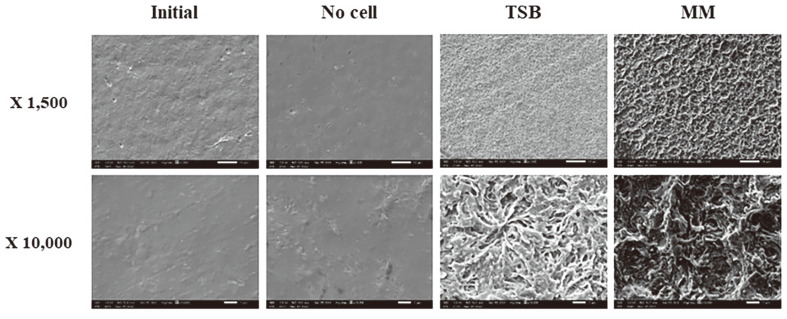
SEM analysis of PHB film surfaces incubated with *B. infantis* PD3 in TSB and MM for 5 d.

Changes in the surface morphology and molecular weight of PHB films were investigated after biodegradation by *B. infantis* PD3 in each medium. Before degradation, the initial PHB film exhibited a smooth surface with no fragments. The surface of the PHB film incubated in TSB for 5 d appeared to be slightly fragmented. The PHB films collected after biodegradation in MM for 5 d were highly fragmented into several small pieces (Fig. 4). Disappearance of the surface gloss of the film was also observed after PHB film degradation, indicating that the surface of the PHB film may have been eroded by PHB depolymerase [27]. SEM images were analyzed to confirm the surface erosion of the PHB film collected after PHB film degradation. The PHB film surfaces were coated with platinum for electron scattering and observed at 1,500× and 10,000× magnification for each experiment (Fig. 5). The surface of the initial PHB film was smooth and exhibited no cracks. The surface became rough and cracked, resulting in unevenness. The surface of the PHB film in the MM was rougher than the surface of the PHB film in the TSB, which was consistent with the result of PHB film degradation in liquid medium (Fig. 2). To further verify the changes in the physical properties of the degraded PHB films, their molecular weights were analyzed using GPC. As shown in Table 2, the average molecular weights of the degraded PHB film were number average (Mn) 37,661 and weight average (Mw) 169,538, which are significantly lower than those of the initial PHB film. When incubated without cells, these numbers changed slightly to Mn 117,859 and Mw 311,160. The polydispersity index (PDI; Mw/Mn) increased from 2.57 to 4.51, suggesting that the molecular structure of the PHB film changed and degraded into molecules of various sizes during degradation by *B. infantis* PD3.

### Potential for Degradation of Other Bioplastics

A halo assay was performed to determine the potential of *B. infantis* PD3 to degrade other bioplastics, such as PBS and PBAT. Unlike PHB, PBS and PBAT are petroleum-based bioplastics. Bioplastics have physical properties that are easily compostable and biodegradable in natural environments [28]. The halo assay was performed using *B. infantis* PD1, PD2, and PD3 in PBS and PBAT double-layer plates containing MM. As shown in Table 4, no halo zone was observed for 5 d in the PBS and PBAT double-layer plates for any of the three strains, indicating that *B. infantis* cannot degrade PBS and PBAT. Thus, we need to consider the possibility that PBS and PBAT require more time to degrade than PHB and that other enzymes are responsible for and specific to PBS and PBAT biodegradation [29, 30].

## Conclusion

PHB is a promising biodegradable plastic; however, effective degradation facilities are required for commercialization. For effective PHB degradation in soil and compost, we isolated three *B. infantis* strains with excellent PHB-degrading abilities from soil. To identify the presence of the PHB-degrading enzyme, *Bacillus* spp. universal primer sets were established, and the presence of *phaZ* and *bdhA* was confirmed using PCR. In addition, the PHB degradation ability improved when the nitrogen source was limited to the medium composition. *B. infantis* PD3 exhibited a high rate of PHB degradation (98.71%). In addition, the degraded PHB film had a decreased molecular weight and surface erosion.

## Figures and Tables

**Table 1:** PCR conditions of PHB degradation enzyme gene.

Step	*phaZ* PCR conditions		*bdhA* PCR conditions	
	Temperature	Time	Temperature	Time
Step 1. Initial denaturation	95°C	5 min	95°C	5 min
Step 2. Denaturation	95°C	1 min	95°C	1 min
Annealing	45–55°C	1 min	54–60°C	1 min
Extension	72°C	40 s	72°C	40 s
	×35 cycle		×35 cycle	
Step 3. Final extension	70°C	5 min	70°C	5 min
Step 4. End hold	8°C	∞	8°C	∞

**Table 2:** Comparison of PHB mass before and after degradation.

	TSB			MM		
	Before	After	PHB degradation rate	Before	After	PHB degradation rate
No cell	0.2069 g	0.2067 g	0.097%	0.1964 g	0.1881 g	4.23%
*B. algicola* SOL02	0.2038 g	0.1982 g	2.72%	0.2152 g	0.2061 g	4.25%
*B. infantis* PD1	0.2054 g	0.2007 g	2.31%	0.1979 g	0.1380 g	30.29%
*B. infantis* PD2	0.2057 g	0.1991 g	3.19%	0.2015 g	0.0919 g	54.41%
*B. infantis* PD3	0.2113 g	0.1978 g	6.39%	0.2028 g	0.0026 g	98.71%

**Table 3:** GPC analysis result of PHB film degradation by *B. infantis* PD3 in MM for 5 d.

	M_n_	M_w_	PDI
Initial	130,374	335,128	2.57
No-cell	117,859	311,160	2.64
*B. infantis* PD3	37,611	169,538	4.51

**Table 4:** Halo zone formation on plates containing various bioplastics.

	P(3HB)	PBS	PBAT
*B. infantis* PD1	+	-	-
*B. infantis* PD2	+	-	-
*B. infantis* PD3	+	-	-
